# Health and Social Care Integration in Scotland: Evidence vs Rhetoric

**DOI:** 10.5334/ijic.7759

**Published:** 2024-01-30

**Authors:** Cam Donaldson, Peter Knight, Alastair L. Noble, Sandy Strathearn

**Affiliations:** 1 Yunus Centre for Social Business & Health, Glasgow Caledonian University, UK; 2 National Centre for Epidemiology & Public Health, Australian National University, Australia; 3 Mean Value Consulting, Formerly NHS Scotland Information Services Division, UK; 4 MBE retired General Practitioner, Nairn, UK; 5 Quality Management, Service Manager for Business Improvement, Perth and Kinross Council, UK

**Keywords:** health, social care, integration, NHS

## Abstract

In this perspective paper we use publicly-available data to show that, despite much positive rhetoric in support of reforms in Scotland to integrate health and social care, these reforms, in their current state, have failed to meet their stated objectives. Rather than regress to the previous system, we propose continued evaluation of even more radical forms of such integration. This analysis, and set of future proposals, are timely given current considerations with respect to a National Care Service in Scotland and recent similar reforms in England and in other countries.

## Introduction

In the UK, integration of health and social care has been a policy objective for over a quarter of a century [1]. Creating a single commissioner across hospital, community and social care services has potential to build services around people’s needs, improving outcomes and value. This approach is being pursued by policy makers in many countries faced with increased prevalence of long-term conditions and associated requirements for access to multi-sectoral care [2].

With the passage of the Health and Care Act (2022), England is embarking on the creation of 42 Integrated Care Systems (ICSs). Thus, it is timely to learn from earlier integration reforms which commenced in Scotland in 2016 [3,4], especially as the integration jurisdictions in England are structured in a similar fashion. For Scotland itself, reflection is opportune given the recent Audit Scotland review of progress [5] and the proposed establishment of a National Care Service (NCS) [6].

Using publicly-available national-level data, progress can be assessed against the aims of integration, to reduce delayed discharges, unnecessary admissions and delays in receiving attention in Accident and Emergency (A&E) Departments as well as improving outcomes in key areas (i.e. mental health and drug-and-alcohol consumption) in which a more-integrated approach to care in community settings would be expected to have a positive impact. The perspectives on interpretation of results and future direction come from a team with extensive experience in social care (SS), primary care practice (AN), evidence and commissioning within health and healthcare (PK) and health economics research (CD). The perspectives are evidence-based.

## Scotland’s innovation in integrated care

A particular form of integration involves taking down financial barriers between different parts of the system. In Scotland, the driver has been to encourage greater involvement of multiple stakeholders (general practitioners, acute clinicians, social workers, nurses, Allied Health Professionals, pharmacists and third sector providers) in local planning of service provision. Across the country, 32 geographically-defined Integration Joint Boards (IJBs) became operational in 2016. IJBs would ostensibly be in charge of two-thirds of the combined NHS and social care budget. The logic is that, by taking down barriers between health and social care budgets, resources can be more-readily moved across the care pathway, allowing care packages to be developed so as to prevent hospital admissions, permit earlier discharge, relieve pressure on A&E and enable a system better placed to tackle Scotland’s mental health, drug and alcohol challenges.

## Integrated care in Scotland: what happened?

Using data from Public Health Scotland, the Scottish Government National Performance Framework, National Records of Scotland (NRS) and the Scottish Health Survey from 2013/14, two years before the legislation was implemented in Scotland, up to the point of the Covid-19 pandemic (2019/20):

- delayed discharges rose from 50,000 to 51,000, with associated hospital inpatient resource costs increasing from £100 m to £140 m;
- unplanned acute admissions rose over the same time period, by 9%, although the number of associated bed days remained fairly static; and
- the percentage of A&E attenders treated within the performance measure of 4-hours maximum declined from 93% to 89%, against a backdrop of nearly 5% more attenders.

Outcome indicators have fared no better, with no improvement in the Warwick-Edinburgh Mental Wellbeing Scale (WEMWBS), similarly so for alcohol-related deaths (1,000 per annum), and with a doubling in the number of drug deaths (rising from nearly 600 to 1200 per annum).

The potential of new integration authorities is tempered by the reality of their limited autonomy. IJBs sit between an influential NHS and democratically-elected councils, many IJB members are non-voting and the Chief Officer (of the IJB and the Health and Social Care Partnership) reports to the Chief Executives of both the NHS and the Council. With funding increasingly constrained, and interests heavily vested in the status quo, proposals to move resources could easily be resisted [7]. The intention was that all unplanned bed days, including delayed discharges, were to be funded from IJB budgets by transfers to Health Boards, such that an increase in bed days would require more funding from IJBs and reductions would require less; the latter allowing any savings to be re-allocated by the IJB to other services [8]. Without some Health Board participation in these (‘set-aside’) arrangements, IJBs had no mechanism for shifting resources if they reduced bed days. In short, cultural barriers and accompanying power relations trumped financial reforms.

## Where now?

After consulting on an NCS, the proposal is that IJBs be reformed in community health and social care boards, funded by Scottish Government on the basis of a resource allocation formula. However, Scotland’s 14 NHS Boards would remain, and run on separate funding arrangements. This would seem to be a backward step. We would propose a move in the opposite direction, strengthening IJBs as a basis for a locality-based approach combined with place-based planning. Budgets could still be allocated to IJBs (or some other form of such) via a formula, but include greater accountability and responsibility for healthcare as well as social care resources. Governance arrangements would need to be strengthened, with, for example, representatives of Professional Health and Social Care Teams and the Third/Charitable Sector appointed with full voting rights. In practical terms, the IJB would be required to determine the right numbers of appropriate staff at locality level, when set against levels of need, budget, other evidence and professional judgement. Key would be to strike the right balance between specialist and generalist care, and drawing on the intelligence available from local and national data. Regarding the latter, data to support locality-based delivery and system-wide planning, spanning both social care and health care utilisation and costs, are available now and are likely to improve as better digital systems are implemented.

## Concluding comments

In line with recent conclusions drawn by the Nuffield Trust [9], evidence from Scotland shows integration of health and social care in its current form, and on its own, is unlikely to achieve stated policy goals. Greater funding has been recommended by several prestigious reviews [10], but with no sign of being acted upon by any major political party. Yet, the promise of a combination of funding uplift and ring-fencing together with more comprehensive locality-empowered integrated care has actually been shown in emergency care and in reshaping care for older people in Scotland [11,12]. If successful on a more-widespread basis, the prize would be treating people closer to home through re-allocating to social care the £1bn Scotland currently spends annually in hospitals for delayed discharges, with similar proportionate gains for England.
